# Aggressive NK-cell leukemia: clinical subtypes, molecular features, and treatment outcomes

**DOI:** 10.1038/s41408-017-0021-z

**Published:** 2017-12-21

**Authors:** Y-T Tang, D Wang, H Luo, M Xiao, H-S Zhou, D Liu, S-P Ling, N Wang, X-L Hu, Y Luo, X Mao, Q-L Ao, J Huang, W Zhang, L-S Sheng, L-J Zhu, Z Shang, L-L Gao, P-L Zhang, M Zhou, K-G Zhou, L-G Qiu, Q-F Liu, H-Y Zhang, J-Y Li, J Jin, L Fu, W-L Zhao, J-P Chen, X Du, G Huang, Q-F Wang, J-F Zhou, L Huang

**Affiliations:** 1 Department of Hematology, Tongji Hospital, Tongji Medical College, Huazhong University of Science and Technology, Wuhan, China; 2 Department of Hematology, Nanfang Hospital, Southern Medical University, Guangzhou, China; 3 Key Laboratory of Genomic and Precision Medicine, Collaborative Innovation Center of Genetics and Development, Beijing Institute of Genomics, Chinese Academy of Sciences, Beijing, China; 4 Department of Pathology, Tongji Hospital, Tongji Medical College, Huazhong University of Science and Technology, Wuhan, China; 5 Institute of Hematology and Blood Diseases Hospital, Chinese Academy of Medical Sciences and Peking Union Medical College, Tianjing, China; 6 Department of Hematology, Peking University Shenzhen Hospital, Shenzhen, China; 7 Department of Hematology, the First Affiliated Hospital of Nanjing Medical University and Jiangsu Province Hospital, Nanjing, China; 8 Department of Hematology, the First Affiliated Hospital, Zhejiang University College of Medicine, Hangzhou, China; 9 Department of Hematology, Beijing Friendship Hospital, Capital Medical University, Beijing, China; 10 Shanghai Institute of Hematology, State Key Laboratory of Medical Genomics, Shanghai Rui Jin Hospital, Shanghai Jiao Tong University School of Medicine, Shanghai, China; 11 Department of Hematology, Southwest Hospital, Third Military Medical University, Chongqing, China; 12 Department of Hematology, Guangdong General Hospital and Guangdong Academy of Medical Sciences, Guangzhou, China; 13 Division of Experimental Hematology and Cancer Biology, Cincinnati Children’s Hospital Medical Center, Cincinnati, OH USA; 14 Division of Pathology, Cincinnati Children’s Hospital Medical Center, Cincinnati, OH USA; 15 University of Chinese Academy of Sciences, Beijing, China; 16 Cancer Biology Research Center, Tongji Hospital, Tongji Medical College, Huazhong University of Science and Technology, Wuhan, China

Aggressive NK-cell leukemia (ANKL) is a rare form of NK cell neoplasm sporadically affecting people from Asia and Central and South America. The median overall survival (OS) is less than 2 months, irrespective of treatments. Epstein-Barr virus (EBV) is mostly detected in the leukemia cells and is proposed to contribute to the pathogenesis of ANKL^1–3^. ANKL represents a distinct disease entity within the continuous spectrum of EBV-associated T/NK-cell lymphoproliferative diseases (EBV-T/NK-LPDs). Patients with ANKL usually manifest a fulminant and extremely aggressive clinical course. However, some clinicopathologic features may be shared with different types of EBV-T/NK-LPDs, which leads to the occurrence of a few cases with features intermediate between two similar disorders^4–6^. The diagnosis of ANKL largely relies on the identification of morphologically and immunophenotypically aberrant leukemia cells^7^. Chromosomal gains and losses, activating *STAT3* and *STAT5* mutations, and *HACE1* hypermethylation have only been sporadically detected^8–10^. Moreover, optimal therapy of ANKL has not yet been established^11^. To date, less than 350 cases of ANKL have been described in English literature worldwide. Because of the rarity of ANKL, the clinical features, potential pathogenesis, therapeutic strategies, and prognostic factors still lack in understanding. A multicenter study is critically needed for better understanding of this disease.

Here we conducted a 13-year retrospective study with 113 confirmed ANKL patients enrolled in 10 clinical centers located in different geographic regions across China. All cases were centrally reviewed by three hematopathologists and three hematologists. Study design, enrolled clinical centers, and data collection were described in the Supplementary Methods. This study was approved by the institutional review board of Tongji Hospital, Tongji Medical College, Huazhong University of Science and Technology. Informed consent was obtained from each individual in accordance with the principles expressed in the Declaration of Helsinki.

From October 2003 to July 2016, a total of 161 suspected cases were collected, and 113 cases with eligibility consensuses after central review were finally enrolled. All the patients were of the Han nationality living in the mainland China and had no history of chronic active EBV disease (CAEBV), severe mosquito bite allergy, hydroa vacciniforme, or other T/NK-LPDs. Patient eligibility and general characteristics, including immunophenotyping and EBV detection of leukemia cells, were summarized in Supplementary Results and Supplementary Tables S1 and S2. The distribution of onset age was illustrated in Supplementary Fig. S1A showing an incidence peak in patients between 21 and 30 years old (29.20%, 33/113), with a male to female ratio of nearly 2:1 in this decade. The median OS was only 55 days (Supplementary Tables S2) and 1-year survival rate was only 4.42% (5/113; Supplementary Fig. S1B), which indicated a dismal outcome of ANKL.

Intriguingly, a subacute clinical course was demonstrated in 18 ANKL patients (15.93%, 18/113). They manifested infectious mononucleosis (IM)-like symptoms (including fever, lymphocytosis or mononucleosis, lymphadenopathy, and hepatosplenomegaly) for more than 90 days (median: 115 days, range: 90–450 days), prior to the fulminant onset (Table 1). Female predominance (*P* = 0.007) was revealed in these patients. Alleviated hyperferritinemia (*P* < 0.001), transaminitis (ALT, *P* = 0.009), and hypofibrinogenemia (*P* = 0.038), suggesting alleviated hemophagocytic lymphohistiocytosis (HLH), liver impairment, and coagulopathy, were also noted at diagnosis in these patients (Table 1 and Supplementary Table S3). A marked survival advantage (*P* < 0.001) was revealed, irrespective of whether or not the patients who received allogeneic hematopoietic stem cell transplantation (allo-HSCT) were excluded (Table 1 and Fig. 1a). After all, allo-HSCT is currently the only treatment modality that can independently improve the survival of ANKL patients^11–13^. Moreover, even if the prolonged prodromal phases were not included in the estimation of the OS of these patients, this marked survival advantage could still be observed in non-transplant patients (*P* = 0.042; Supplementary Fig. S2). All these data suggested that patients with prolonged prodromal phases, whom we define here as “subacute ANKL”, may represent a clinical subtype of ANKL which differed from the others, whom we define here as “classic ANKL”.

**Table 1:** Comparison of clinical characteristics based on clinical subtypes

Characteristics	Subacute ANKL *N*=18 (range)	Classic ANKL *N*=95 (range)	*P*
Gender, no. of patients (%)			**0.007**
Male	5 (27.78)	59 (62.11)	
Female	13 (72.22)	36 (37.89)	
Median ALT, U/l (range)	69.35 (12.00–311.00)	108.00 (6.00–933.00)	**0.009**
Median ferritin, mg/l (range)	618.00 (184.00–2280.58)	4330.00 (99.00–146,000.00)	**<0.001**
Median fibrinogen, g/l (range)	2.27 (0.50–5.01)	1.51 (0.24–5.48)	**0.038**
TP53 mutation, no. of patients (%)^a^			**0.038**
Positive	0 (0.00)	11 (37.93)	
Negative	8 (100.00)	18 (62.07)	
Median prodromal period, days (range)	115 (90–450)	15 (0–79)	**<0.001**
OS, days (range; all patients)	213.5 (98–551)	49 (8–1480)	**<0.001**
OS, days (range; non-transplant patients)^b^	206 (98–551)	44 (8–273)	**<0.001**

*N* number, *ALT* alanine aminotransferase, *OS* overall survival

^a^ *TP53* mutation screened in eight subacute ANKL patients and 29 classic ANKL patients

^b^When patients received allo-HSCT were excluded, there were 17 subacute ANKL patients and 89 classic ANKL patients in the study

Bold: statistically significant

**Fig. 1:**
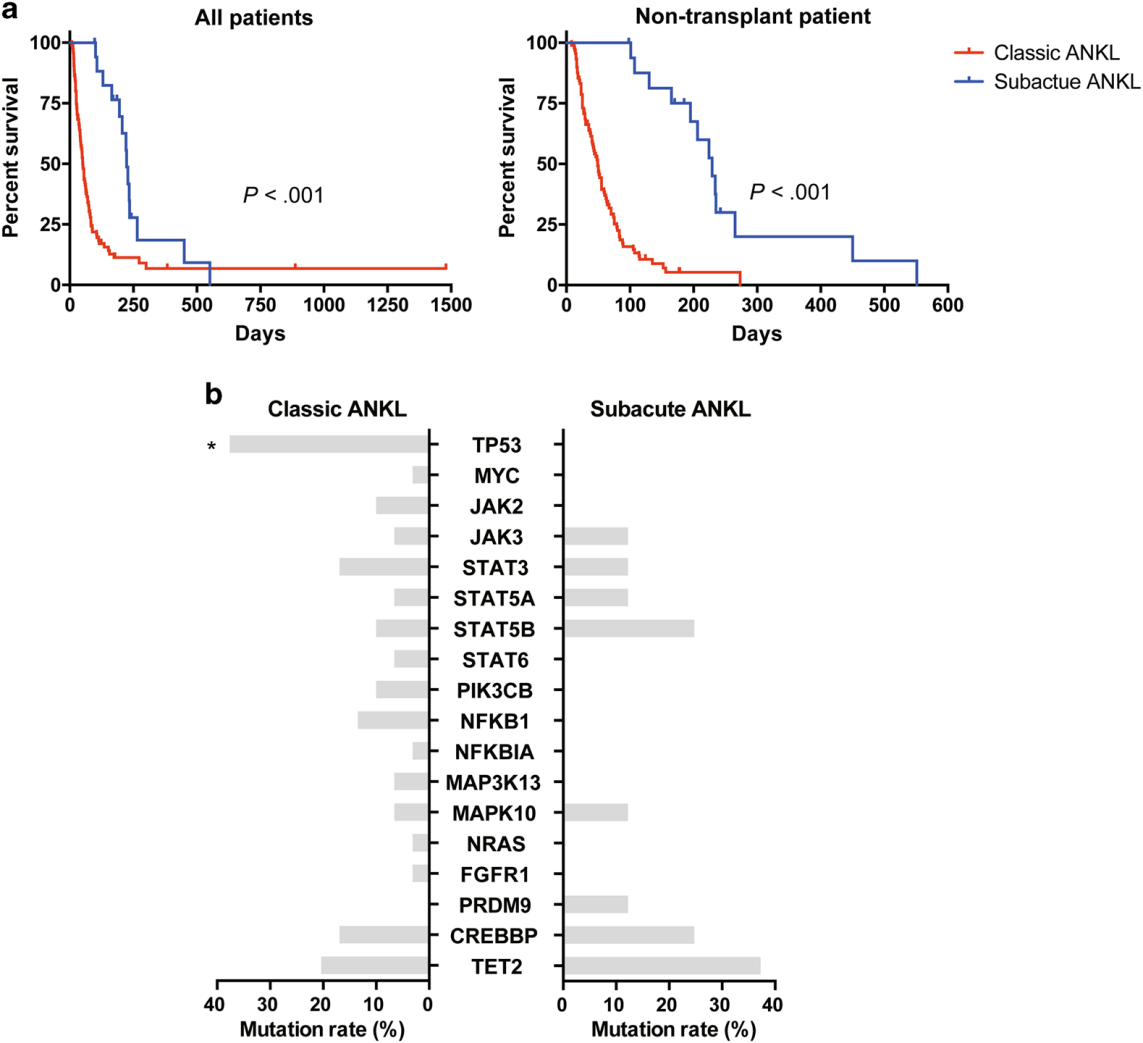
Outcomes and mutational patterns of ANKL subtypes **a** Comparison of overall survival (OS) between subacute ANKL patients (*N* = 18) and classic ANKL patients (*N* = 95). OS was estimated from the onset of disease to the date of death or the end of the study. A marked survival advantage was revealed in subacute ANKL patients (left, *P* < 0.001), even if the patients receiving allo-HSCT (*N* = 7) were excluded (right, *P* < 0.001). **b** Somatic mutations identified by targeted sequencing in eight subacute ANKL patients and 29 classic ANKL patients were shown. The custom sequencing panel contained 18 candidate genes, including transcriptional factors (*TP53* and *MYC*), JAK-STAT pathway genes (*JAK2*, *JAK3*, *STAT3*, *STAT5A*, *STAT5B*, and *STAT6*), other signaling pathway genes (*PIK3CB*, *NFKB1*, *NFKBIA*, *MAP3K13*, *MAPK10*, *NRAS,* and *FGFR1*), and epigenetic regulators (*PRDM9*, *CREBBP,* and *TET2*). Gene mutation patterns were similar between two subtypes, while *TP53* gene mutations enriched in classic ANKL patients

To clarify the underlying pathogenesis of the two clinical subtypes, genes of interest were screened by Ion Torrent AmpliSeq™ using a custom sequencing panel in 37 ANKL patients, including 8 subacute ANKL patients and 29 classic ANKL patients. The panel contained 18 candidate genes (Fig. 1b) identified in our previous whole-genome sequencing analysis of eight ANKL patients, including transcriptional factors, JAK-STAT pathway genes, other signaling pathway genes, and epigenetic regulators. The sequencing depth of these samples was more than 2000×. Our results showed that the *TP53* gene had a significant lower mutation rate in subacute ANKL than that in classic ANKL (*P* = 0.038; Fig. 1b and Supplementary Table S4). Except for that, the other gene mutation patterns including genes in the JAK-STAT pathway were similar between the two groups, suggesting that the key driving mechanism is still similar between the two subtypes of ANKL. Notably, *TP53* mutations were not found in patients of subacute ANKL subtype (Table 1 and Fig. 1b), while enriched in 11 classic ANKL patients (37.93%, 11/29; Supplementary Fig. S3). This result was consistent with the relatively moderate clinical course and improved survival for subacute ANKL patients.

The treatment decision for each patient was made at each clinical center after careful assessment. Since there is no standardized initial treatment for ANKL, chemotherapeutic regimens varied. CHOP-like (containing anthracycline and vincristine), L-ASPA-based (SMILE, AspaMetDex, L-GemOx, and L-ASPA plus dexamethasone)^14, 15^, and HLH-04 regimens (containing dexamethasone and etoposide) were conducted in this study. Seven patients were subjected to allo-HSCT with myeloablative conditioning regimen when they achieved CR after chemotherapy (CHOP-like, *n* = 1; L-ASPA-based, *n* = 6). The median time from diagnosis to allo-HSCT was 73 days (range: 38–128 days). The clinical characteristics of patients in each subgroup were summarized in Supplementary Table S5, and no differences between each subgroups were revealed. The median follow-up time was 55 days (range: 8–1480 days) for the entire cohort and 887 days (range: 384–1480 days) for 3 survivors.

Patients receiving allo-HSCT exhibited significantly superior survivals when compared to the others without allo-HSCT (*P* < 0.001). The median OS was 300 days (range: 174–1480 days) and 2-year OS rate was 42.86% (3/7; Supplementary Fig. S4A). Further subgroup analysis for patients receiving chemotherapy alone revealed significant OS benefit achieved only in patients treated with L-ASPA-based chemotherapy (*n* = 19, *P* = 0.008; Supplementary Fig. S4B). Of the 19 patients with L-ASPA-based chemotherapy alone, 13 patients received a median of two (range: 1–4) cycles of AspaMetDex. The median OS was 115 days (range: 37–450 days). As induction therapy, the CR and overall response rates (ORR) of AspaMetDex in newly diagnosed patients was 30.77% (4/13) and 76.92% (10/13), respectively. Reduction of plasma EBV DNA copies and ferritin levels were observed in nine (69.23%) patients, which was in accordance with the ORR. Grade 3 and Grade 4 hematologic adverse events were common and were recorded in 7 (53.84%) patients. Five of them had severe infection. Meanwhile, damage of liver function was rare (7.69%, 1/13) and no patient died of regimen-related side effects.

We evaluated a variety of clinical features and therapeutic strategies as possible factors that of prognostic significance. Univariate analysis revealed the following clinical factors to be significantly associated with shorter survival: thrombocytopenia (<30 × 10^9^/l), elevated serum LDH level (>800 IU/l), hypoalbuminemia (<35 g/l), hyperferritinemia (>1500 IU/l), classic ANKL, treatment without L-asparagine-based chemotherapy, or allo-HSCT (Supplementary Table S6). Multivariate analysis rendered elevated serum LDH level (>800 IU/l), clinical subtype, treatment (administration of L-asparagine-based chemotherapy, and allo-HSCT) to be valuable predictors of survival (Supplementary Table S7).

In the present study, we systemically explored the features of this deadly type of NK-cell neoplasms. Especially, a subtype of ANKL with clinical and molecular characteristics was depicted. According to the clinicopathological evolution of EBV-T/NK-LPDs^5^, we propose to define these patients as “subacute ANKL”. Although this subtype resembles CAEBV-transformed ANKL (EBV-T/NK- LPDs category A3)^6^ in some aspects of clinical presentations, they are different in onset age and clinical course^4, 5^. CAEBV is an indolent disease primarily affecting children and young adults^4^. While ANKL usually presents a relatively acute disease mainly occurs in the middle-aged population, even though a subacute clinical course may manifest in a subset of patients. By identifying the group of subacute ANKL patients, clinicians should be alert to those ANKL patients presenting only IM-like symptoms during their prolonged prodromal phases, since early diagnosis and timely treatment before they evolve into aggressive phases is critical for survival. In addition, AspaMetDex was revealed to be an effective and well-tolerated initial treatment in ANKL patients. Multivariate analysis in our study showed that the administration of allo-HSCT was one of the major factors affecting survival (HR = 0.022, 95% CI: 0.005–0.097). Accordingly, the administration of AspaMetDex as induction therapy, SMILE as post-remission consolidation therapy^13^ and allo-HSCT as finally curative therapy^12^ could be a promising treatment strategy for ANKL patients. The presence of bias can hardly be avoided, due to the retrospective nature of our study plus the rarity of ANKL. However, these data depicted the clinical features and molecular characteristics of ANKL, and furthermore provided insights into the treatments and outcomes of this deadly type of leukemia.

## Electronic supplementary material

Supplementary Information
