# Supplementary material for: Aggressive NK-cell leukemia: clinical subtypes, molecular features, and treatment outcomes
Source: Blood Cancer J. 2017 Dec 21;7(12):660. doi: 10.1038/s41408-017-0021-z (PMC5802497; doi:10.1038/s41408-017-0021-z)
Supplement: Supplementary file 1 — Supplementary Information [file 41408_2017_21_MOESM1_ESM.pdf]

Supplement to ***Yu-ting Tang, et al. Aggressive NK-Cell leukemia: clinical subtypes, molecular features and treatment outcomes.***

This Supplementary Information has been provided by the authors to give readers additional information about their work.

SUPPLEMENTARY INFORMATION

CONTENTS:

SUPPLEMENTARY METHODS 3
Study design 3
Enrolled clinical centers and data collection 4
Immunophenotyping 4
Genomic DNA extraction and TCR clonality assays 5
Detection of EBV 5
Fluorescence-activated cell sorting of leukemia cells 5
Mutational Analysis 6
Accession code 7
Statistical analysis 7
URLs 8
SUPPLEMENTARY RESULTS 9
Patient eligibility and clinical samples 9
General characteristics of 113 enrolled ANKL patients 9
SUPPLEMENTARY TABLES 11
Table S1. Clinical characteristics of 113 enrolled ANKL patients 11
Table S2. General clinical characteristics of the entire cohort 15
Table S3. Comparison of clinical characteristics based on clinical subtypes 16
Table S4. Comparison of the mutation frequencies of 18 genes between subacute and classic ANKL patients 17
Table S5. Clinical characteristics of patients in each treatment subgroup 18
Table S6. Univariate analysis of OS using the Cox regression model 19
Table S7. Multivariate analysis of OS using the Cox regression model 20
SUPPLEMENTARY FIGURES 21
Figure S1. Onset age distribution and overall survival 21
Figure S2. Comparison of OS between subacute and classic ANKL patients 22
Figure S3. *TP53* gene structure and mutation sites identified in classic ANKL patients.... 23
Figure S4. Overall Survival of ANKL patients receiving different treatment strategies. ... 24
SUPPLEMENTARY REFERENCES 25

SUPPLEMENTARY METHODS

Study design

Ten clinical centers in China participated in this retrospective multicenter study. The eligibility criteria were made according to the World Health Organization classification for tumors of the hematopoietic and lymphoid tissues.[1] The inclusion criteria were defined as: (1) an extremely aggressive clinical course usually presenting fever, pancytopenia, coagulopathy, lymphadenopathy, hepatosplenomegaly, and rapid progression to disseminated intravascular coagulation (DIC), hemophagocytic lymphohistiocytosis (HLH) and multiple organ failure within days to weeks; (2) infiltration of atypical medium to large-sized lymphocytes containing broad, pale or slightly basophilic cytoplasm, prominent azurophilic granules and bizarre nucleus in the peripheral blood (PB), bone marrow (BM) and affected tissues; (3) leukemia cells of NK cell origin confirmed by flow cytometry or immunohistochemistry (IHC); (4) germline configuration of T-cell receptor (TCR) genes. The infection of EBV is routinely assessed in the study, but not included as an inclusion criteria, since EBV-negative ANKL, which is pathologically and clinically indistinguishable from EBV-positive cases, was reported previously.[2, 3] Exclusion criteria were as follows: (1) pathologically defined extranodal NK/T-cell lymphoma (ENKTL), blastic plasmacytoid dendritic cell neoplasm, hepatosplenic and cutaneous γδT-cell lymphoma and other peripheral T-cell lymphoma; (2) Cases expressing clonal TCRαβ or TCRγδ on leukemia cells confirmed by flow cytometry or IHC. All cases were centrally reviewed by three hematopathologists and three hematologists.

This study was approved by the institutional review board of Tongji Hospital, Tongji Medical College, Huazhong University of Science and Technology. Informed consent was obtained from

each individual in accordance with the principles expressed in the Declaration of Helsinki.

Enrolled clinical centers and data collection

Ten clinical centers in China participated in this retrospective multicenter study, including Tongji Hospital Affiliated to Tongji Medical College of Huazhong University of Science and Technology, Institute of Hematology and Blood Diseases Hospital Affiliated to Chinese Academy of Medical Sciences & Peking Union Medical College, the First Affiliated Hospital of Nanjing Medical University and Jiangsu Province Hospital, the First Affiliated Hospital of Zhejiang University College of Medicine, Nanfang Hospital Affiliated to Southern Medical University, Peking University Shenzhen Hospital, Beijing Friendship Hospital Affiliated to Capital Medical University, Shanghai Rui Jin Hospital Affiliated to Shanghai Jiao Tong University School of Medicine, Southwest Hospital Affiliated to the Third Military Medical University, Guangdong General Hospital and Guangdong Academy of Medical Sciences.

We collected clinical data regarding age, gender, clinical manifestation at diagnosis, treatment and follow-up. Laboratory data recorded included PB and BM examination, immunophenotype of leukemia cells, T-cell receptor (TCR) gene configuration, tissue pathology and EBV infections.

Immunophenotyping

Leukemia cells of NK cell origin were confirmed by IHC or flow cytometry. Flow cytometry was performed on BM aspiration samples. IHC was performed on BM trephine biopsy specimens and formalin-fixed, paraffin-embedded (FFPE) tissue samples. All the methods were performed as previously.[4]

Genomic DNA extraction and TCR clonality assays

For the detection of EBV-DNA, T-cell receptor (TCR) clonality and mutational analysis, genomic DNA was extracted from BM or affected tissues, including fresh BM mononuclear cells, FACS-sorted leukemic NK cells at diagnosis, or preserved BM aspirate smears and FFPE tissue sections using suitable DNA Extraction Kits following the manufacturer's instructions. TCR clonality was determined by multiplex PCR according to European BIOMED-2 protocol as described previously.[4, 5]

Detection of EBV

EBV-DNA in plasma and FACS-sorted leukemic NK cells was assessed by quantitative real-time polymerase chain reaction (EBV-qPCR). For FFPE sections of affected tissues, EBV infection of leukemia cells was determined by *in situ* hybridization using EBER oligonucleotides (EBER-ISH) and IHC. All the methods were performed as described previously.[4, 6]

Fluorescence-activated cell sorting of leukemia cells

For EBV infection analysis, leukemia cells from ANKL patients were isolated by fluorescence-activated cell sorting (FACS). Briefly, fresh bone marrow (BM) from healthy donors or ANKL patients was prepared with Lymphoprep™ (Axis-Shield, Oslo, Norway) by gradient centrifugation to obtain mononuclear cells (MNCs). The MNCs were then washed with phosphate-buffered saline (PBS) and stained with PE-conjugated anti-CD56 antibody (BD Biosciences, San Jose, CA, USA) and PerCP-conjugated anti-CD3 antibody (BD Biosciences, San Jose, CA, USA). The antibodies were incubated with the cells for 30 min at 4°C. The MNCs

were then sorted by FACSAria (BD Biosciences, San Jose, CA, USA). For most cases, leukemia cells were isolated by sorting $CD56^{+}CD3^{-}$ cells. The purity of the FACS-sorted cells was confirmed by flow cytometry and was over 99%.

Mutational Analysis

As for the detection of somatic mutation, only samples with the percentage of leukemia cells over 10% were included, which were assessed by flow cytometry. The concentration and quality of extracted genomic DNA was assessed by NanoDrop spectrophotometer (Thermo Fisher Scientific) and gel electrophoresis, respectively. Ten nanogram of genomic DNA was used for library preparation using Ion AmpliSeq Library kits 2.0 (Life Technologies). Sequencing was performed as previously.[5] We aligned AmpliSeq reads using the Burrows-Wheeler Aligner algorithm (BWA-MEM).[7] Reads were kept with no more than three mismatches and mapping qualities no less than 20 after removing the redundant reads. For the identification of somatic mutations in leukemia cells, we merged the bam files of two granulocyte cell samples into a "combined" normal control to identify somatic mutations. Then, we detected nonsynonymous single-nucleotide variants (SNVs) with CASpoint using the following criteria: (i) The sequencing depth of the tumor and normal samples must be larger than 100. (ii) The minimum read numbers of the supporting mutant allele in the tumor samples must be more than 20, and the read numbers of the supporting mutant allele in the normal samples must be no more than 3 or no more than 10 if the mutant allele of the normal sample is different from that of the tumor sample. (iii) Sequencing reads supporting the mutant allele must contain both positive and negative strands. (iv) Sequencing reads supporting the alleles that are neither reference bases nor mutated bases account for no more than 0.1 of all reads covering the site. (v) The mutant

allele frequency of SNVs in the tumor samples must be no more than 40% due to the low purity of tumor cells in bone marrow. (vi) SNVs listed in dbSNP build 138 were removed.[8] Finally, SNVs occurring in two or more samples were removed unless the same variation appeared in the COSMIC database v76.[9]

Accession code

The raw sequence data reported in this paper have been deposited in the Genome Sequence Archive[10] in BIG Data Center[11], Beijing Institute of Genomics (BIG), Chinese Academy of Sciences, under accession numbers CRA000344 that are publicly accessible at http://bigd.big.ac.cn/gsa.

Statistical analysis

Overall survival (OS) was estimated from the onset of disease to the date of death or the end of the study. To eliminate the contribution of prolonged prodromal phases to the survival advantages of subacute ANKL patients, we excluded the periods of prodromal phases from the estimation of OS in ANKL patients in Supplementary Figure S2. Complete remission (CR) was defined as the complete resolution of all known disease on imaging along with a negative BM aspiration and biopsy. Partial remission (PR) was defined as improved laboratory findings, symptoms, and imaging but detectable minimal residual leukemia cells in BM.[12] The analysis of categorical variables was performed using Fisher's exact test for 2×2 tables or Pearson's $\chi 2$ test. Student's *t* test and the Mann-Whitney U test were applied to continuous variables. Comparisons of mean values for clinical characteristics among different treatment subgroups were carried out by one-way analysis of variance. Univariate and multivariate models using

Cox regression analysis were employed to assess the hazard ratios of patient characteristics on survival. Differences in survival were compared using the log-rank test and estimated by the Kaplan-Meier method. All calculations were performed using SPSS software version 16.0 (SPSS, Chicago, IL). *P* values less than 0.05 (two-tailed) were considered to be statistically significant.

URLs

COSMIC database, http://cancer.sanger.ac.uk/cancergenome/projects/cosmic/;

dbSNP, http://www.ncbi.nlm.nih.gov/projects/SNP/;

Beijing Institute of Genomics: http://bigd. big.ac.cn/gsa.

SUPPLEMENTARY RESULTS

Patient eligibility and clinical samples

From October 2003 to July 2016, a total of 161 suspected cases were collected in this study, 113 cases (Supplementary Table S1) with eligibility consensuses after central review were finally enrolled. Leukemia cells were detected on BM smears in all patients with an average percentage of 18.55% (median 11.00%, range: 3.50-76.00%) of nucleated cells. The immunophenotype was assessed by flow cytometry. The leukemia cells do not express surface CD3 (94.69%, 107/113), while express cytoplasmic CD3 (50.00%, 29/58), CD56 (92.04%, 104/113), CD16 (64.29%, 63/98), CD2 (97.09%, 100/103) and CD7 (66.07%, 74/112) (Supplementary Table S1). Germline configuration of T-cell receptor (TCR) genes had been confirmed in all the samples from the 113 patients. In addition, 7 patients were biopsied (LN, n=2; spleen, n=2; liver, n=3; skin, n=1) and diagnosed as ANKL by histopathology and immunohistochemistry (IHC). Plasma EBV-DNA was measured in 98 patients by quantitative real-time polymerase chain reaction (EBV-qPCR) with a median load of 3.18×10^{6} copies/mL (range: 4.47×10^{2} - 2.06×10^{8} copies/mL). Moreover, EBV infection status was further confirmed in FACS-sorted leukemic NK cells from 35 patients by EBV-qPCR and on formalin-fixed, paraffin-embedded (FFPE) tissue sections from 7 patients by *in situ* hybridization using EBER oligonucleotides (EBER-ISH) (Supplementary Table S1).

General characteristics of 113 enrolled ANKL patients

The following described general characteristics were summarized in Supplementary Table S2. Most patients presented with sudden fever (94.69%, 107/113) at diagnosis. Splenomegaly and hepatomegaly were detected by ultrasonography in 91.51% (97/106) and 70.89 % (56/79) of

the patients, respectively, and 39.77% (35/88) had superficial lymphadenopathy. Pancytopenia was common in these patients. Leukopenia, anemia and thrombocytopenia were identified in 65.49% (74/113), 70.80% (80/113) and 88.50% (100/113) of the patients, respectively. Whereas 15.04% (17/113) of the patients had leukocytosis (median 18.90, 10.90 - 57.37, $\times 10^9$/L). Transaminitis and hypoalbuminemia were identified in about 90%, and hyperbilirubinemia in 60% of the patients. However, the levels of serum transaminases (ALT, median 93.00 U/L; AST, median 128.00 U/L) and total bilirubin (TBIL, median 27.00 μmol/L) were only slightly or moderately above normal in half of the patients. Markedly increased concentrations of lactate dehydrogenase (LDH, median 735.50 U/L), caused by cell destruction, were identified in nearly all patients (98.11%, 104/106). Prominent hypofibrinogenemia (median 1.57 g/L) and hyperferritinemia (median 2961.00 μg/L), associated with HLH, were identified in 45.10% (46/102) and 87.50% (70/80) of the patients, respectively. Although NK-cell activity and soluble CD25 (sCD25) assay were not readily available at all enrolled clinical centers, HLH was determined in 93.41% (85/91) of the patients according to HLH-2004 diagnostic criteria.[13]

SUPPLEMENTARY TABLES

Table S1. Clinical characteristics of 113 enrolled ANKL patients

No.	*Gender*	*Age (years)*	*WBC (×10^9/L)*	*HB (g/L)*	*PLT (×10^9/L)*	*ALT (U/L)*	*AST (U/L)*	*LDH (U/L)*	*TBIL (μmol/L)*	*ALB (g/L)*	*Ferritin (μg/L)*	*Fibrinogen (g/L)*	*Immunophenotype*	*Plasma EBV-DNA (copies/mL)*	*EBV Infection in leukemia cells*	*Duration of prodromal phases (days)*	*Treatment*	*OS (days)*
4	Male	58	21	131	13	57	72	547	33.4	28.5		1.78	sCD3-cyCD3-CD56+CD2+CD7-CD16-CD8-		Pos (Sorting-PCR)	14	CHOP-like	35
15	Female	45	3.66	97	97	12	13	600	12.5	29.1		5.01	sCD3-CD56+CD2+CD7+CD16-CD8-CD94+CD161+	25500000	Pos (Sorting-PCR)	90	supportive	101
17	Female	15	2.13	78	63.6	12	33	442	26.2	34.4	342	3.87	sCD3-CD56+CD2+CD7+CD16+CD8-CD94+CD161+	770000	Pos (Sorting-PCR)	17	supportive	41
19	Female	27	57.37	63	71	27.6	149.2	4837	12.7	35.4	1708.4	1.357	sCD3-CD56+CD2+CD7+CD16+CD8-CD57-	124179	Pos (Sorting-PCR)	35	supportive	65
20	Female	30	5.69	64	49	196	59	909	9.71	30.2	2525		sCD3-CD56+CD2+CD7+CD16-CD8-CD57-	236000	Pos (EBER-ISH) (Liver), Pos (Sorting-PCR)	30	CHOP-like+Allo-HSCT	174
21	Male	13	2.05	71	21	34	28	220	15.6	24.5	1311	2.19	sCD3-CD56+CD2+CD7+CD16+CD8+CD57-CD94+	722000	Pos (Sorting-PCR)	20	HLH-04	124
22	Male	59	1.3	71	23	114	187	334	148	28	1500	1	sCD3-CD56-CD2+CD7+CD16+CD57-CD94+		Pos (Sorting-PCR)	25	L-ASPA based	156
23	Male	38	7.74	87	47	191	121			34.8			sCD3-cyCD3-CD56+CD2+CD7+CD16-CD8-		Pos (Sorting-PCR)	106	L-ASPA based	224
25	Female	48	0.5	74	17	36	42	651	14.8	26.5	1897	1.11	sCD3-cyCD3-CD56+CD2+CD7+CD16-CD8-CD94+	34000	Pos (Sorting-PCR)	9	L-ASPA based	37
26	Female	25	4.7	124	58.1	54	33	500	29.7	39.3	184	2.93	sCD3-cyCD3-CD56+CD2+CD7-CD16+CD8-CD94+	193000	Pos (Sorting-PCR)	348	L-ASPA based	450
27	Female	15	0.85	90.7	47.9	218	144	1125	12.9	33.5	4059	1.59	sCD3-cyCD3+CD56+CD2+CD7+CD16+CD8+CD94+CD161+CD158a/h+CD158b+CD158e-	447	Pos (Sorting-PCR)	15	L-ASPA based	80
28	Male	22	3.37	113	85	48	63	789	12.2	32.9	422	2.8	sCD3-cyCD3-CD56+CD2-CD7+CD16+CD8-CD94+CD161+CD158a/h-CD158b-CD158e-	50000000	Pos (Sorting-PCR)	12	supportive	27
29	Male	25	0.84	100	15	140	260		19.2	25.6		0.67	sCD3-CD56+CD2+CD7+CD16-CD8-CD57-CD94+		Pos (Sorting-PCR)	20	CHOP-like	83
31	Male	24	5.28	101	36	84	134	1514	98.5		20204	0.55	sCD3-CD56+CD2+CD7+CD16-CD8-CD94+CD161+	20200000	Pos (Sorting-PCR)	15	L-ASPA based	55
32	Male	26	4.15	110	33	475	707	2054	46.7	31.3	19506	1.03	sCD3-CD56+CD2+CD7+CD16+CD8-CD94+CD161+CD158a/h+CD158b-CD158e+	206000000	Pos (Sorting-PCR)	20	L-ASPA based	84
41	Male	39	1.06	136	25.3	200	216	646	113	27.5		3.06	sCD3-cyCD3-CD56+CD2+CD7+CD16-CD8-CD94+	35000000		8	supportive	17
42	Male	42	1.02	76.8	17.8	18	132		6.5	20	32300	1.69	sCD3-CD56+CD2+CD7+CD16+CD8-CD94+		Pos (Sorting-PCR)	14	supportive	23
43	Male	53	6.71	115	96	42	48	583	8.9	29.4	3510	1.25	sCD3-CD56+CD2+CD7+CD16-CD8-CD94+CD161+	16700000		15	supportive	25
44	Male	38	1.15	115	86.3	13	35	703	18.4	33.8			sCD3-CD56+CD2+CD7-CD16-CD8+CD57-CD94+CD161+	693000		15	L-ASPA based+Allo-HSCT	887
45	Male	52	2.06	119	32	301	363	1087	78.2	26.8		0.61	sCD3-CD56+CD2+CD7-CD16-CD57+CD94+CD161+	1770000		39	supportive	46
46	Male	27	4.11	134	91.1	27	49	525	11	36.3		4.33	sCD3-cyCD3-CD56+CD2+CD7-CD16-CD8-	8080000	Pos (EBER-ISH) (LN), Pos (Sorting-PCR)	8	L-ASPA based+Allo-HSCT	1480
47	Female	55	12.86	88	13	51	164	1158	15.3	20.1		0.5	sCD3-CD56+CD2+CD7+CD16-CD158a/h-CD158b+CD158e+		Pos (Sorting-PCR)	4	supportive	23
48	Male	25	2.43	108	22.7	306	513	3925	43.3	25.8	108000	1.65	sCD3-cyCD3-CD56+CD2+CD7+CD16-CD8-CD94+	3120000		11	HLH-04	16
49	Female	59	4.6	91	103	23	24	286	3.5	26		3.98	sCD3-CD56+CD2+CD7+CD161+CD158a/h-CD158b+CD158e-	2650000		135	supportive	170
50	Female	47	20.2	96.2	40	98	147	4695	60.4	32.7		1.58	sCD3-CD56-CD7+CD16+CD57-CD94+		Pos (Sorting-PCR)	13	supportive	18
51	Female	25	16.32	78	93	44	131	436	76.3	15.5	494.6	0.54	sCD3-CD56+CD2+CD7+CD16+CD8-CD94+	10000000	Pos (Sorting-PCR)	135	supportive	229
52	Male	26	2.42	91	5	189	128	875	22.6	31	46784	0.82	sCD3-CD56+CD2+CD7+CD8-CD94+	920000		35	CHOP-like	51
53	Female	15	15.45	110	108	412	580	2700	113	22	15000	2.54	sCD3-CD56+CD2+CD7+CD16+CD8+CD57-CD94+	39000000		25	CHOP-like	55
54	Male	61	35.65	122.4	69.2	122	204	236	191.6	21.5	6166	1.4	sCD3-cyCD3-CD56-CD2+CD7+CD16-CD8-CD57-CD94+CD158a/h-CD158b-CD158e-	4420000		0	L-ASPA based	178
56	Male	25	2.73	76	71	78.7		614.5	40.6	40		2.15	sCD3-cyCD3-CD56+CD2+CD7-CD16+	125000		115	L-ASPA based+Allo-HSCT	221
57	Female	32	1.6	105	39	223	332	980	11	37.9	618	2.5	sCD3-CD56+CD2+CD7-CD16+CD8-	7400000		450	supportive	551

58	Male	65	4.3	153	58	54	84	404	21	33.7	2918	1.1	sCD3-cyCD3+CD56+CD2+CD7-CD16-CD8-	3240000		20	supportive	44
59	Male	36	2.5	119	99	49	68	484	10	37	491	1.6	sCD3-CD56+CD2-CD7+CD16+CD8-	437000		90	supportive	98
60	Male	39	3.6	129	52	466	218	483	253	28.8	5133.6	2.4	sCD3-CD56+CD2+CD7+CD16+CD8-	2890000		17	supportive	62
61	Male	78	5.2	99	20	42	163		68	29.7	6532	0.8	sCD3-CD56+CD2-CD7+CD16+CD8-	13600000		10	supportive	12
62	Male	28	1.6	77	51	48	62	1154	14	36	1971	2.4	sCD3-CD56+CD2+CD7+CD16+CD8-	37400		28	CHOP-like	49
63	Female	25	5.4	101	80	34	58	324	14	20.7	2608	1.2	sCD3-cyCD3+CD56+CD2+CD7+CD16+CD8-	4710000		40	supportive	50
64	Male	33	2.6	68	26	17	37	572	39	21.1		0.7	sCD3-CD56+CD2+CD7+CD16+CD8+CD57-CD94+	261000		10	supportive	20
65	Male	55	0.98	130	12	63	201	1726	11.4	31.3	58300	0.5	sCD3-CD56+CD2+CD7+CD16+CD8+CD94+CD158a/h-CD158b+CD158e+		Pos (Sorting-PCR)	7	supportive	25
66	Male	15	3.01	96	17	86		887	41	22.7	1700	1.26	sCD3-CD56+CD2+CD7+CD16-CD8+CD94+	9860000		11	HLH-04	13
67	Male	35	1.33	81	22	68.1	80.2	152	35.2	22.3	710.5	1.802	sCD3-CD56+CD2+CD7+CD16-CD8+CD57-CD161+	64700000		30	supportive	74
101	Female	35	1.24	95	22	141	548	9844	25.6	22.2		1.03	sCD3-CD56+CD2+CD7+CD16+CD8-	78200000		6	HLH-04	8
103	Male	54	3.32	88	58	33	44	596	7.3	25.7	2330	4.32	sCD3-CD56+CD2+CD7+CD8-	5630000		15	supportive	29
109	Female	27	2.88	74	29	72	463	1867	21.5	24.6	438.6	0.94	sCD3+CD56+CD2+CD7+CD8-CD57-CD64-CD94+CD161+CD158a/h-CD158b-CD158e-	673000	Pos (EBER-ISH) (SP), Pos (Sorting-PCR)	4	HLH-04	17
110	Male	22	2	98	30	181	692	1867	187.7	25	8000	1.27	sCD3-CD56+CD2+CD7+CD8+CD16+CD57-CD94+		Pos (Sorting-PCR)	10	L-ASPA based	124
111	Female	20	5.82	102	55	311	75	311	143.6	32.7	447.2	2.97	sCD3-cyCD3+CD56+CD2+CD7-CD16+CD8-	540000	Pos (Sorting-PCR)	90	L-ASPA based	242
130	Female	31	1.81	67.2	24	45	64	554	33.9	30.7		3.21	sCD3-CD56+CD7+CD16+CD8-	42900000	Pos (EBER-ISH) (Liver, SP)	7	supportive	33
131	Male	29	4.6	116	55	933	1552	436	40.1	21.7	4330	1.4	sCD3-cyCD3+CD56+CD2+CD7+CD16+CD8+	73700000		70	L-ASPA based	273
132	Male	30	2.81	117	62	48	74	791	29.2	25.1	146000	1.03	sCD3-cyCD3+CD56+CD7+CD8-	164000		6	CHOP-like	41
133	Male	47	2.8	128.8	53	165	117	768	11.3	27.6	44800	1.4	sCD3-cyCD3+CD56+CD2+CD7+CD16+CD8+	71800	Pos (Sorting-PCR)	9	CHOP-like	107
134	Male	24	2.02	84	26	110	48	406	8.7	25.4	6070	1.55	sCD3-cyCD3+CD56-CD2+CD7+CD16-CD8-		Pos (Sorting-PCR)	5	CHOP-like	37
135	Female	50	2.59	122	107	57	131	1218	11.8	24.4		2.1	sCD3-CD56+CD2+CD7+CD8-CD16-	78300000		20	CHOP-like	59
136	Female	42	0.29	101	4	427	76	410	51.6	33.7		2.3	sCD3-cyCD3+CD56+CD2+CD7-CD16-CD8-	5400000		30	supportive	49
137	Female	26	1.25	80.6	31	6	34	900	9.8	31.5		1.78	sCD3-CD56+CD7+CD8-		Pos (EBER-ISH) (LN)	13	supportive	40
138	Female	26	1.8	76	20		47	600				3.4	sCD3-CD56+CD2+CD7+CD8-	6590000		21	supportive	30
139	Male	14	16.7	105	88	77	104	773	7.88	35.7	1147	0.99	sCD3-cyCD3+CD56-CD2+CD7-CD16+CD8-	13300000		15	CHOP-like+Allo-HSCT	174
140	Female	27	2.6	93	39	458	476	2280	129	26.9	59380	1.6	sCD3-CD56+CD2+CD16+CD8-	10500000	Pos (EBER-ISH) (Liver), Pos (Sorting-PCR)	19	L-ASPA based	75
141	Male	40	24.1	77	288	365	192	2557	191.2			0.24	sCD3-cyCD3+CD56+CD2+CD7+CD16+CD8-	27400000		0	supportive	23
142	Female	22	0.98	75.2	26	10	8	262	7.4	20.3		1.35	sCD3-cyCD3-CD56-CD2+CD7+CD8+		Pos (Sorting-PCR)	40	supportive	52
143	Female	19	3.18	91.3	24	226	481	1062	60.1	15	9310	0.5	sCD3-CD56+CD2+CD7+CD8+CD16+CD94+	235000		60	supportive	63
144	Female	44	5.44	96	384	181	274	502	7.8	31.4	341.2	5.48	sCD3-cyCD3+CD56+CD2+CD7+CD16+CD8+CD94+	8340000		35	CHOP-like	114
145	Male	37	1.85	103	63	108	134	592	123	23.2		1.53	sCD3-CD56+CD2+CD7+CD16-	56300		60	supportive	79
146	Male	22	3.3	134	68	254	617	2150	17	34.9		1.07	sCD3+cyCD3+CD56+CD2+CD7-CD16+CD8+CD94+	3340000		10	CHOP-like	25
147	Male	50	2.5	147	35	67	63	684	5	34.3	40000	1.8	sCD3-CD56+CD7+CD8-	4350000		14	CHOP-like	38
148	Female	14	18.9	103	16	24	95		105	21		2.32	sCD3-CD56+CD2+CD7-CD16-CD8-	2490000		220	CHOP-like	234
149	Male	22	10.9	79	130	45.6	54	266	7.6	23.2	1289	2.76	sCD3-cyCD3+CD56+CD2+CD7+CD16+CD8+	256000		33	HLH-04	89
150	Female	42	4.1	80.9	13	32	54	424	14	37	245	1.5	sCD3-CD56+CD2+CD7+CD16+CD8+	557000		10	supportive	55
151	Male	30	2.39	87	24	30	41		16.9	25.5	7530	2.67	sCD3-CD56+CD2+CD7+CD16-	17400000		15	supportive	28
152	Male	50	3.73	98.2	116	38	57	500	8.5	26.9		3.2	sCD3-CD56+CD2+CD7+CD8-CD16-	7280000		18	L-ASPA based	43
153	Male	25	2.77	90	51	83	48	387	16.3	29.8		1.46	sCD3-cyCD3+CD56+CD2+CD7-CD16+CD8+	91300000		115	supportive	130
154	Female	42	1.7	95	64	347	591	958	33.3	33.6	1105	0.8	sCD3+cyCD3+CD56+CD2+CD7-CD16+CD8-CD94+	696000	Pos (Sorting-PCR)	40	CHOP-like	55
155	Male	30	1.66	134	19	137	99	632	12.6	27.2	12400	3.02	sCD3-CD56+CD2+CD7-CD8-	96400		13	supportive	31
156	Male	22	2.07	99.4	63.9	17	49		20.7	27.4	7660	1.82	sCD3-CD56+CD2+CD7+CD16-CD8+		Pos (Sorting-PCR)	65	supportive	88

157	Female	50	12.7	102	25	16	24	225	8.7	34.5	99	2.31	sCD3-cyCD3+CD56+CD2+CD7+CD16+CD8+	380000		30	supportive	70
158	Male	58	4.99	143	60	93	163	1762	187	24.7	7940	2.25	sCD3-CD56+CD2+CD7+CD16+CD8-	5730		5	supportive	15
159	Female	46	1.86	137	43.6	101	332	1563	27	25.4		0.5	sCD3-CD56+CD2+CD7+CD16+CD8+CD94+	2870000		220	supportive	235
160	Female	40	5	98	45	66	150	532	12.3	24			sCD3-cyCD3+CD56+CD2+CD7+CD16-CD8+	957000		180	HLH-04	195
161	Female	42	1.76	68	3	327	533	563	282	21.2	8250	0.93	sCD3-cyCD3+CD56+CD2+CD7+CD16+CD8-	3620000		20	L-ASPA based	48
162	Female	38	12.6	69	181	243.5	737.5	2744	253.9	26.6	12300	1.8	sCD3-cyCD3+CD56+CD2+CD7+CD16+CD8+	979000		27	HLH-04	50
163	Male	37	54.3	95	42	32	100	608	10	26	983	2.42	sCD3-cyCD3-CD56+CD2+CD7+CD16-CD8-	13200		40	supportive	83
164	Female	17	3.3	72	22	94	338	6639	71.8	19.6		0.645	sCD3-cyCD3+CD56+CD7-CD16+CD8-	4500000		11	supportive	15
165	Female	76	26.8	147	133	77	286	1556	131	29.7	2250	0.5	sCD3-cyCD3+CD56+CD2+CD7-CD16+CD8+	72300000		135	supportive	265
166	Female	54	6.26	99	51	23	332	344	7.4	29.9	722	1.32	sCD3-cyCD3-CD56+CD2+CD7+CD16-CD8-	1560000		90	supportive	206
167	Male	38	2.1	101	56	370	529	1340	151	23.5	1500	1.31	sCD3-cyCD3+CD56+CD7-CD16+CD8-	1460000		49	supportive	60
168	Male	52	0.45	99	14	109	362	3153	72.4	25.6	95350.6	1.51	sCD3-cyCD3-CD56+CD2+CD7+CD16-CD8-	1180000	Pos (Sorting-PCR)	7	HLH-04	62
169	Female	48	2.11	95.8	60.9	70	69	236	26.1	28.4		2.45	sCD3-CD56+CD2+CD7-CD16-CD8+		Pos (EBER-ISH) (Skin)	15	L-ASPA based	115
170	Male	45	2.07	90	57	125		435.9	34.1	26.5	545.84		sCD3+cyCD3-CD56+CD2+CD7-CD16+CD8-	14000000	Pos (Sorting-PCR)	7	L-ASPA based+Allo-HSCT	384
171	Male	46	2.14	109	15	58	171	1679	27.8	28	11077	1.61	sCD3-cyCD3+CD56+CD2+CD7+CD8-	1250000		14	CHOP-like	64
172	Male	16	4.02	99.2	31	452	1425	2224	80.6	23.4	15001	0.86	sCD3-cyCD3-CD56+CD2+CD7+CD16-CD8-	37300000		29	CHOP-like	70
173	Female	60	4.9	59	10.4	13	19	230	24.2	21	1314	4.69	sCD3-cyCD3-CD56+CD2+CD7+CD16-CD8-	854000		90	CHOP-like	185
174	Male	59	6.03	69	56	69	328	2005	725	22.9	5297	1.22	sCD3-cyCD3-CD56+CD2+CD7+CD16-CD8-	208000		20	HLH-04	75
175	Female	74	2.16	77	12	126.2		528	98.7	23.3	6358.69		sCD3-CD56-CD2+CD7-CD16+CD8-CD94+	4150000		20	CHOP-like	27
176	Male	21	2.73	118	152	230.3	67	1309	25.5	33.4	2280.58	2.21	sCD3-cyCD3+CD56+CD2+CD7-CD16+CD8+	1150000		105	HLH-04	165
177	Female	50	3.43	71	8	272		2180.6	19.6	32.8	2100	1.362	sCD3-cyCD3-CD56+CD2+CD7-CD16+	77100		15	CHOP-like	18
178	Male	69	3.84	64	265	198		2574	55	30			sCD3-cyCD3-CD56+CD2+CD7-CD16+	6400000		15	L-ASPA based	105
179	Female	33	2.53	100	91	129		1360	31	33.9	31000	4.4	sCD3-cyCD3-CD56+CD2+CD7-	4820000		7	HLH-04	14
180	Male	37	0.95	46	3	55		102.6	13.8	33.2	2000	0.96	sCD3-cyCD3-CD56+CD2+CD7-CD16+	3530000		79	L-ASPA based+Allo-HSCT	300
181	Male	34	3.58	67	6	105.6	115.3	1467	60.1	16.5	2000	1.21	sCD3-cyCD3+CD56+CD2+CD7-CD16+	38100000		14	CHOP-like	16
182	Male	35	2.72	107	74	410		560	49	28	2000		sCD3+cyCD3-CD56+CD2+CD7-CD16+CD8-	370000		10	supportive	30
183	Female	58	3.41	110	44	451		953	27.3	27	3004		sCD3-cyCD3-CD56+CD2+CD7-CD16+	521000		11	supportive	25
184	Male	63	1	51	166	556		2100	41.4	29	3674		sCD3-cyCD3-CD56+CD2+CD7-CD16+	341000		60	supportive	67
185	Female	37	3.24	78	42	72.7		2356	144.5	20		4.2	sCD3-cyCD3-CD56+CD2+CD7-CD16+	4330000		100	CHOP-like	107
186	Female	63	1.8	96	61			2000	56	26	2000	4.1	sCD3-CD56-CD2+CD7+CD16+CD8-	7120000		35	CHOP-like	135
187	Male	10	2.9	111	90	126		980	21	31			sCD3-cyCD3+CD56+CD2+CD7-CD16+	580000		15	supportive	35
188	Female	30	6.13	115	82	345		580	33.6	27.9	2700	1	sCD3-CD56-CD2+CD7+CD8-CD16+	3400000		5	L-ASPA based	25
189	Male	47	2.16	134	72	259.2		2100	21.4	24.9	2000	2.37	sCD3-cyCD3+CD56+CD2+CD7-CD16+	1410000	Pos (Sorting-PCR)	15	supportive	29
190	Female		11	89	23	126		457	27	27	2000	4.56	sCD3-cyCD3-CD56+CD2+CD7-CD16+CD8+	30700000		7	HLH-04	21
191	Male	66	1.9	113	39	66	78	480	19	26.5	9921	1.9	sCD3-CD56+CD7+CD8-	458000		10	supportive	40
192	Male	44	26.7	136	12	844	37	1233	28	28.6	40000	1.5	sCD3-CD56+CD7+CD8-	22500000		30	supportive	42
193	Male	56	1.9	114	133	179	428	1051	304	28.6	40000	1.6	sCD3-cyCD3+CD56+CD7-CD8-	5700000		15	supportive	23
194	Female	27	0.62	78	3	251		560	28.6	20.6	40000	1.31	sCD3+cyCD3-CD56+CD2+CD7-CD16+CD8-	2410000		10	HLH-04	16
195	Female	70	0.76	93	52	194	170	446	21.2	29.8	10363	1.01	sCD3-cyCD3-CD56+CD2+CD7-CD16+CD8-		Pos (Sorting-PCR)	40	L-ASPA based	177
196	Male	23	2.61	80	30	66	186	1867	5.6	55.1	12066	1.01	sCD3-CD56+CD2+CD7+CD16-CD8+	51700000	Pos (Sorting-PCR)	30	L-ASPA based	152

Abbreviations

No.: number; WBC: white blood cell; HB: hemoglobin; PLT: platelet; ALT: alanine aminotransferase; AST: aspartate aminotransferase; LDH: lactate dehydrogenase; TBIL: total bilirubin; ALB: albumin; Pos: positive; Sorting-PCR: EBV-DNA copies of FACS-sorted leukemia cells measured by quantitative real-time polymerase chain reaction; EBER-ISH: EBV infection determined by in situ hybridization using EBER oligonucleotides; LN: lymph node; SP: spleen; Allo-HSCT: allogeneic hematopoietic stem cell transplantation; HLH: hemophagocytic lymphohistiocytosis; L-ASPA: L-asparaginase; OS: overall survival.

Table S2. General clinical characteristics of the entire cohort

Characteristics	*Median (range)*	*No. of patients available*	*No. of abnormal patients (%)*
Gender			
Male		64	
Female		49	
Age, years	37.00 (10 - 78)	112	
Fever		113	107 (94.69)
Splenomegaly		106	97 (91.51)
Hepatomegaly		79	56 (70.89)
Lymphadenopathy		88	35 (39.77)
HLH		91	85 (93.41)
WBC, $\times 10^9$/L	2.80 (0.29 - 57.37)	113	74 (< 4.00, 65.49)<br>17 (> 10.00, 15.04)
HB, g/L	97.00 (46.00 - 153.00)	113	80 (< 110.00, 70.80)
PLT, $\times 10^9$/L	45.00 (3.00 - 384.00)	113	100 (<100.00, 88.50)
ALT, U/L	93.00 (6.00 - 933.00)	111	92 (> 33.00, 82.88)
AST, U/L	128.00 (8.00 - 1552.00)	95	89 (> 32.00, 93.68)
LDH, U/L	735.50 (102.60 - 9844.00)	106	104 (> 214.00, 98.11)
TBIL, μmol/L	27.00 (3.50 - 725.00)	111	68 (> 20.50, 61.26)
ALB, g/L	27.45 (15.00 - 55.10)	110	100 (< 35.00, 90.91)
Fibrinogen, g/L	1.57 (0.24 - 5.48)	102	46 (< 1.50, 45.10)
Ferritin, μg/L	2961.00 (99.00 - 146000.00)	80	70 (> 500.00, 87.50)
Plasma EBV-DNA, copies/mL	3.18×10^6<br>(4.47×10^2 - 2.06×10^8)	98	62 (> 1×10^6, 63.27)
OS, days	55 (8-1480)	113	5 (> 365, 4.42)

Abbreviations are explained in Supplementary Table S1.

Table S3. Comparison of clinical characteristics based on clinical subtypes (continued*)

Characteristics	*Subacute ANKL* N=*18*	*Classic ANKL* N=*95*	*P*
Median Age, years (range)	36.50 (14.00 - 76.00)	37.00 (10.00 - 78.00)	0.866
Median WBC, $\times 10^9$/L (range)	4.65 (1.60 - 26.80)	2.60 (0.29 - 57.37)	0.657
Median HB, g/L(range)	98.50 (59.00 - 147.00)	96.00 (46.00 - 153.00)	0.559
Median PLT, $\times 10^9$/L (range)	53.00 (10.40 - 152.00)	39.00 (3.00 - 384.00)	0.431
Median AST, U/L (range)	85.00 (13.00 - 332.00)	132.00 (8.00 - 1552.00)	0.168
Median LDH, U/L (range)	516.00 (230.00 - 2356.00)	790.00 (102.60 - 9844.00)	0.156
Median TBIL, μmol/L (range)	25.50 (3.50 - 144.50)	27.15 (5.00 - 725.00)	0.627
Median ALB, g/L (range)	29.75 (15.50 - 40.00)	27.10 (15.00 - 55.10)	0.330

Abbreviations are explained in Supplemental Table S1.

* Supplementary to Table 1.

Table S4. Comparison of the mutation frequencies of 18 genes between subacute and classic ANKL patients

Genes	*Number of patients carrying mutation*		*P*
	Subacute ANKL N=*8*	*Classic ANKL* N=*29*	
CREBBP	2	5	0.619
FGFR1	0	1	0.594
JAK2	0	3	0.343
JAK3	1	2	0.607
MAP3K13	0	2	0.445
MAPK10	1	2	0.607
MYC	0	1	0.594
NFKB1	0	4	0.266
NFKBIA	0	1	0.594
NRAS	0	1	0.594
PIK3CB	0	3	0.343
PRDM9	1	0	0.054
STAT3	1	5	0.747
STAT5A	1	2	0.607
STAT5B	2	3	0.283
STAT6	0	2	0.445
TET2	3	6	0.326
TP53	0	11	**0.038**

Table S5. Clinical characteristics of patients in each treatment subgroup

Characteristics	*Supportive* N=*51*	*HLH-04* N=*14*	*CHOP-like* N=*22*	*L-ASPA based* N=*19*	*P*
Gender					0.968
Male	29	7	12	10	
Female	22	7	10	9	
Age, years	39	27	39.5	30	0.309
Fever	47	14	21	18	0.717
Splenomegaly	41	11	22	17	0.324
Hepatomegaly	17	9	13	12	0.583
Lymphadenopathy	14	4	7	8	0.769
HLH	34	13	18	16	0.604
*WBC, ×10^9/L**	2.77	2.81	2.81	3.73	0.661
*HB, g/L**	99.40	92.00	97.60	95.80	0.264
*PLT, ×10^9/L**	44.00	26.00	33.00	47.90	0.995
*ALT, U/L**	64.50	117.50	110.00	191.00	0.222
*AST, U/L**	91.50	345.00	122.50	170.00	0.045
*LDH, U/L**	608.00	1334.50	1056.00	571.50	0.115
*TBIL, µmol/L**	21.20	27.80	26.00	43.40	0.234
*ALB, g/L**	28.00	24.55	25.80	28.20	0.147
*Fibrinogen, g/L**	1.60	1.65	1.61	1.27	0.135
*Ferritin, µg/L**	2608.00	3788.79	6214.35	6616.00	0.493
*Plasma EBV-DNA, copies/mL**	2.88×10^6	1.17×10^6	3.34×10^6	5.41×10^6	0.315

* Median value of each test.

Abbreviations are explained in Supplemental Table S1.

Table S6. Univariate analysis of OS using the Cox regression model

Characteristics	*Hazard ratio*	*95% CI*	*P*
Age, >40 years	1.329	0.877 - 2.014	0.180
WBC, >3×10^9/L	0.962	0.640 - 1.444	0.850
HB, >90g/L	0.731	0.477 - 1.119	0.149
PLT, > 30×10^9/L	0.531	0.344 - 0.818	**0.004**
ALT, >200U/L	1.544	0.986 - 2.418	0.058
LDH, >800U/L	1.882	1.230 - 2.879	**0.004**
TBIL, >20.5μmol/L	1. 377	0.889 - 2.132	0.152
ALB, >35g/L	0.307	0.130 - 0.723	**0.007**
Fibrinogen, > 1g/L	0.831	0.505 - 1.368	0.467
Ferritin, >1500mg/L	2.810	1.478 - 5.342	**0.002**
Plasma EBV-DNA, >3×10^6 copies/mL	1.237	0.799 - 1.915	0.341
Subacute ANKL	0.327	0.182 - 0.588	<**0.001**
L-ASPA based regimens	0.453	0.251 - 0.819	**0.009**
CHOP-like regimens	0.916	0.532 - 1.577	0.752
HLH-04 regimens	1.165	0.610 - 2.224	0.643
Allo-HSCT	0.112	0.034 - 0.371	<**0.001**

Abbreviations are explained in Supplemental Table S1.

Table S7. Multivariate analysis of OS using the Cox regression model

*Characteristics**	*Hazard ratio*	*95% CI*	*P*
PLT, > 30×10⁹/L	0.681	0.417 - 1.111	0.124
LDH, >800U/L	1.753	1.104 - 2.783	**0.017**
ALB, >35g/L	0.437	0.162 - 1.176	0.101
Subacute ANKL	0.087	0.035 - 0.217	<**0.001**
Treatments			
Supportive	-	-	-
HLH-04 regimen	0.762	0.385 - 1.508	0.435
CHOP-like regimens	0.578	0.312 - 1.074	0.083
L-ASPA based regimens	0.187	0.088 - 0.399	<**0.001**
Allo-HSCT	0.022	0.005 - 0.097	<**0.001**

Abbreviations are explained in Supplemental Table S1.

* Hyperferritinemia was excluded in the multivariate analysis due to the limited data availability of serum ferritin levels (70.80%, 80/113).

SUPPLEMENTARY FIGURES

Figure S1. Onset age distribution and overall survival

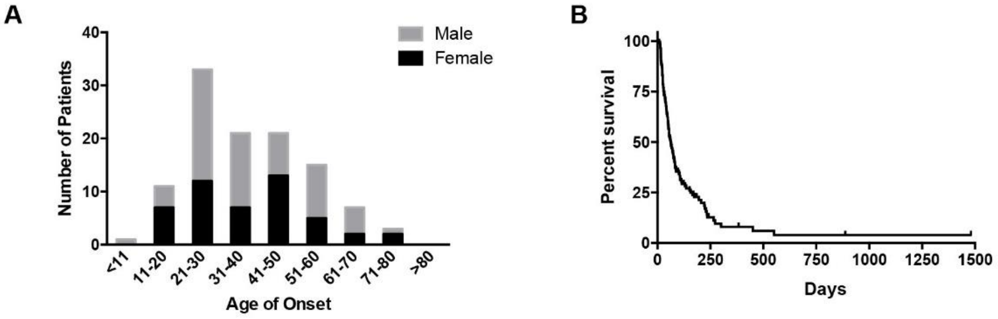

Figure S1. (A) Numbers of male (grey) and female (black) patients with onset ages within the indicated ranges were shown in bars. (B) Overall survival of 113 enrolled ANKL patients.

Figure S2. Comparison of OS between subacute and classic ANKL patients

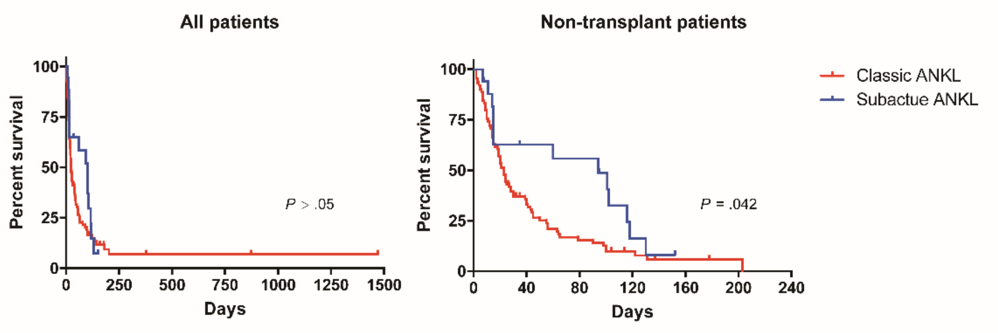

Figure S2. Comparison of OS between subacute (N=18) and classic (N=95) ANKL patients. OS was estimated from the diagnosis of ANKL to the date of death or the end of the study. To eliminate the contribution of prolonged prodromal phases to the survival advantages of subacute ANKL patients, we excluded the periods of prodromal phases from the estimation of OS in ANKL patients in this figure. Marked survival advantage could still be observed in non-transplant patients (P = 0.042).

Figure S3. ***TP53*** **gene structure and mutation sites identified in classic ANKL patients**

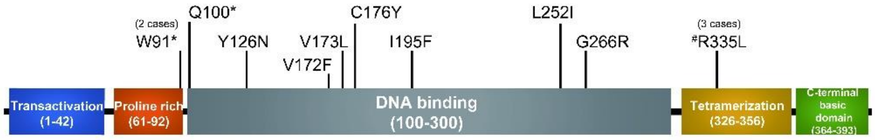

Figure S3. 13 *TP53* gene mutations were identified in 11 classic ANKL patients. Most of the detected *TP53* missense mutations were located in the DNA binding domain (61.54%, 8/13), while two recurrent mutations (5/13) are located in proline rich domain and tetramerization domain, respectively. Among the mutations located in DNA binding domain, four (p.V173, p.C176, p.I195, p.G266) had been found to mutate in other tumors[14, 15] and two (p.V172F, p.V173L) had been validated to be loss-of-function mutations[14]. Loss-of-function mutations located in the DNA binding domain in *TP53* gene hand been frequently identified in various malignancies and related to disease progression and worse survival.[5, 14, 16-19]

Figure S4. Overall Survival of ANKL patients receiving different treatment strategies.

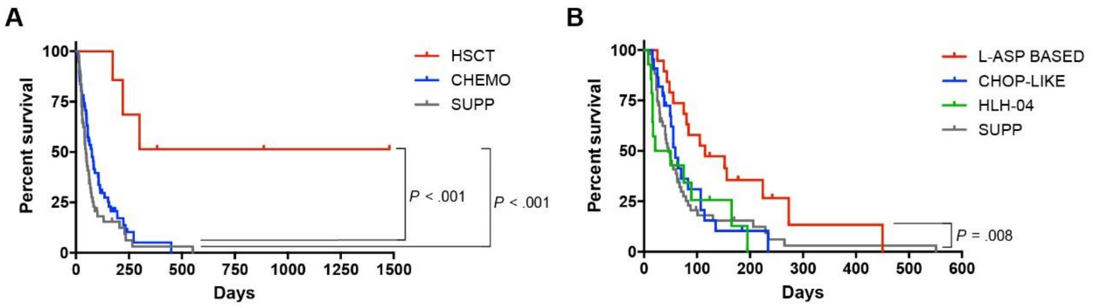

Figure S4. (A) Comparison of OS in ANKL patients receiving allo-HSCT (red, N=7) and other therapeutics, including chemotherapy alone (CHEMO, blue, N=55) and supportive care (SUPP, grey, N=51). The difference in survival was compared between each two of the three groups. In contrast, the median OS of patients administered with CHEMO and patients received SUPP were only 64 days (range: 8 – 450 days) and 40 days (range: 12 - 551 days), respectively. No differences in OS between CHEMO and SUPP groups were observed. *P*-values with statistical significance were shown in the figure. (B) Further subgroup analysis for patients in CHEMO group revealed that the median OS of patients receiving L-ASPA based (red, N=19), CHOP-like (blue, N=22) and HLH-04 regimens (green, N=14) was 94.5 days (range: 25 - 273 days), 51 days (range: 16 - 135, days) and 19 days (range: 8 - 124 days), respectively. When compared with SUPP group (Grey, N=51), significant OS benefit was achieved only in patients treated with L-ASPA based chemotherapy (n=19, $P = 0.008$), but not in patients treated with CHOP-like (n=22, $P = 0.610$) or HLH-04 (n=14, $P = 0.611$) regimens. *P*-value with statistical significance was shown in the figure.

SUPPLEMENTARY REFERENCES

1. Swerdlow SH, Campo E, Pileri SA, Harris NL, Stein H, Siebert R, *et al.* The 2016 revision of the World Health Organization classification of lymphoid neoplasms. *Blood* 2016 May 19; **127**(20)**:** 2375-2390.
2. Nicolae A, Ganapathi KA, Pham TH, Xi L, Torres-Cabala CA, Nanaji NM, *et al.* EBV-negative Aggressive NK-cell Leukemia/Lymphoma: Clinical, Pathologic, and Genetic Features. *Am J Surg Pathol* 2017 Jan; **41**(1)**:** 67-74.
3. Gao J, Behdad A, Ji P, Wolniak KL, Frankfurt O, Chen YH. EBV-negative aggressive NK-cell leukemia/lymphoma: a clinical and pathological study from a single institution. *Mod Pathol* 2017 May 26.
4. Li C, Tian Y, Wang J, Zhu L, Huang L, Wang N, *et al.* Abnormal immunophenotype provides a key diagnostic marker: a report of 29 cases of de novo aggressive natural killer cell leukemia. *Transl Res* 2014 Jun; **163**(6)**:** 565-577.
5. Cao Y, Zhu T, Zhang P, Xiao M, Yi S, Yang Y, *et al.* Mutations or copy number losses of CD58 and TP53 genes in diffuse large B cell lymphoma are independent unfavorable prognostic factors. *Oncotarget* 2016 Dec 13; **7**(50)**:** 83294-83307.
6. Kimura H, Ito Y, Kawabe S, Gotoh K, Takahashi Y, Kojima S, *et al.* EBV-associated T/NK-cell lymphoproliferative diseases in nonimmunocompromised hosts: prospective analysis of 108 cases. *Blood* 2012 Jan 19; **119**(3)**:** 673-686.
7. Li H, Durbin R. Fast and accurate long-read alignment with Burrows-Wheeler transform. *Bioinformatics* 2010 Mar 01; **26**(5)**:** 589-595.
8. Li MM, Datto M, Duncavage EJ, Kulkarni S, Lindeman NI, Roy S, *et al.* Standards and Guidelines for the Interpretation and Reporting of Sequence Variants in Cancer: A Joint Consensus Recommendation of the Association for Molecular Pathology, American Society of Clinical Oncology, and College of American Pathologists. *J Mol Diagn* 2017 Jan; **19**(1)**:** 4-23.
9. Forbes SA, Beare D, Gunasekaran P, Leung K, Bindal N, Boutselakis H, *et al.* COSMIC: exploring the world's knowledge of somatic mutations in human cancer. *Nucleic Acids*

Res 2015 Jan; **43**(Database issue)**:** D805-811.

10. Wang Y, Song F, Zhu J, Zhang S, Yang Y, Chen T, *et al.* GSA: Genome Sequence Archive. *Genomics, proteomics & bioinformatics* 2017 Feb; **15**(1)**:** 14-18.

11. Members BIGDC. The BIG Data Center: from deposition to integration to translation. *Nucleic Acids Res* 2017 Jan 04; **45**(D1)**:** D18-D24.

12. Hamadani M, Kanate AS, DiGilio A, Ahn KW, Smith SM, Lee JW, *et al.* Allogeneic Hematopoietic Cell Transplantation for Aggressive NK Cell Leukemia. A Center for International Blood and Marrow Transplant Research Analysis. *Biol Blood Marrow Transplant* 2017 May; **23**(5)**:** 853-856.

13. Ramos-Casals M, Brito-Zeron P, Lopez-Guillermo A, Khamashta MA, Bosch X. Adult haemophagocytic syndrome. *Lancet* 2014 Apr 26; **383**(9927)**:** 1503-1516.

14. Hainaut P, Pfeifer GP. Somatic TP53 Mutations in the Era of Genome Sequencing. *Cold Spring Harb Perspect Med* 2016 Nov 01; **6**(11).

15. Freed-Pastor WA, Prives C. Mutant p53: one name, many proteins. *Genes Dev* 2012 Jun 15; **26**(12)**:** 1268-1286.

16. Robles AI, Jen J, Harris CC. Clinical Outcomes of TP53 Mutations in Cancers. *Cold Spring Harb Perspect Med* 2016 Sep 01; **6**(9).

17. Holmfeldt L, Wei L, Diaz-Flores E, Walsh M, Zhang J, Ding L, *et al.* The genomic landscape of hypodiploid acute lymphoblastic leukemia. *Nat Genet* 2013 Mar; **45**(3)**:** 242-252.

18. Cancer Genome Atlas N. Comprehensive molecular portraits of human breast tumours. *Nature* 2012 Oct 04; **490**(7418)**:** 61-70.

19. Cancer Genome Atlas Research N. Integrated genomic analyses of ovarian carcinoma. *Nature* 2011 Jun 29; **474**(7353)**:** 609-615.
